# Peripheral serotonin regulates postoperative intra-abdominal adhesion formation in mice

**DOI:** 10.1038/s41598-017-10582-w

**Published:** 2017-08-30

**Authors:** Jianbin Bi, Simin Zhang, Zhaoqing Du, Jia Zhang, Yan Deng, Chang Liu, Jingyao Zhang

**Affiliations:** 1 Department of Hepatobiliary Surgery, The First Affiliated Hospital of Xi’an Jiaotong University, Xi’an Shaanxi, 710061 People’s Republic of China; 2 Institute of Advanced Surgical Technology and Engineering, the First Affiliated Hospital of Xi’an Jiaotong University, Xi’an, 710061 Shaanxi Province China; 3 Department of SICU, The First Affiliated Hospital of Xi’an Jiaotong University, Xi’an Shaanxi, 710061 People’s Republic of China

## Abstract

The aim of the present study is to investigate the role and potential mechanisms of peripheral serotonin in postoperative intra-abdominal adhesion formation in mice. The caecum-rubbing operations were conducted for intra-abdominal adhesion formation modelling in wild-type and Tph1−/− mice. The deficiency of serotonin significantly decreased the adhesion scores, weight loss, and adhesion thickness as well as levels of collagen fibres and hydroxyproline in the adhesive tissues. The Tph1−/− mice exhibited a milder inflammatory response and oxidative stress in the adhesive tissues than did the wild-type mice. Moreover, the deficiency of serotonin reduced the levels of PAI-1 and fibrinogen, and raised the t-PA and t-PA/PAI levels in the peritoneal fluids. Moreover, the expressions of CD34, VEGF, TGF-β and 5-HT_2B_ receptor in the adhesive tissues were significantly decreased in the Tph1−/− group mice. Furthermore, the Tph1−/− +5-HTP group showed more severe adhesions than did the Tph1−/− group mice, and the p-chlorophenylalanine (PCPA) could markedly alleviated the adhesion formation in the WT mice. In conclusion, the present study showed that peripheral serotonin regulated postoperative intra-abdominal adhesion formation by facilitating inflammation, oxidative stress, disorder of the fibrinolytic system, angiopoiesis and TGF-β1 expression via the 5-HT_2B_ receptor in the adhesive tissues.

## Introduction

Postoperative intra-abdominal adhesion formation is the most common complication after intra-abdominal surgery. The incidence rate reaches 90% to 95% with limited preventive measures^1^. The adhesions may cause intestinal obstruction, intra-abdominal pain, infertility and increase subsequent operation difficulty^2, 3^. The pathogenesis of postoperative intra-abdominal adhesion formation is limited. The most acceptable opinion is that the injury of the peritoneum or tissues and the subsequent wound healing processes generate the adhesion^4^. Numerous studies have verified disorders of the fibrinolytic process, such as the imbalance of tissue-type plasminogen activator (t-PA) and plasminogen activator inhibitor-1 (PAI-1), which contribute to adhesion formation^5, 6^. Angiogenesis-related factors, such as vascular endothelial growth factor (VEGF), CD31, and CD34, have been demonstrated to be highly expressed in adhesive tissues^7, 8^. Moreover, the inflammatory process plays a vital role in the pathogenesis of postoperative intra-abdominal adhesions^9^. Several anti-inflammatory drugs have been developed to alleviate postoperative intra-abdominal adhesion formation^10–12^. Additionally, hypoxia performs an important role in adhesion formation after injury. Hypoxia stimulates a cascade of events that leads to oxidative stress, nitrative stress and lipid peroxidation, which further fuel inflammation and cause direct injury to aggravate adhesion formation^13, 14^. In addition, several proteins including transforming growth factor β1 (TGF-β1), cyclo-oxygenases (COXs), matrix metalloproteases (MMPs), and tissue inhibitors of metalloproteases(TIMPs) are associated with adhesion formation^15^.

Serotonin, or 5-hydroxytryptamine (5-HT), is a vital vasoactive substance and regulatory factor in the gastrointestinal (GI) tract in addition to its role as a brain neurotransmitter. Tryptophan hydroxylase 1 (Tph1) is the rate-limiting enzyme in peripheral serotonin synthesis. According to Bader *et al*., Tph1 knockout mice maintained approximately a 4% serotonin level in the duodenum; however, they maintained other normal conditions similar to the wild-type C57BL/6 mice^16, 17^. Peripheral serotonin participates in the regulation of platelet aggregation, cardiac function, metabolism, immune responses, and other functions^18–21^.

Peripheral serotonin plays important roles in many diseases. It has been verified that serotonin has marked pro-inflammatory and pro-oxidation functions in inflammation-linked diseases^22–24^. Serotonin facilitates tissue remodelling and fibrosis by up-regulating TGF-β1 expression^25, 26^. In addition, the dysregulation of peripheral serotonin is involved in the pathogenesis of cardiac remodelling, sepsis, diabetes, colitis, pulmonary arterial hypertension, ischaemia reperfusion injury, liver regeneration, tumourigenesis and other conditions^27–30^. However, there was no evidence in previous studies that serotonin regulates postoperative intra-abdominal adhesion formation. The purpose of the present study was to explore the impact of serotonin on tissue fibrosis, the fibrinolytic system, angiogenesis and the inflammatory response in postoperative intra-abdominal adhesion formation in mice.

## Results

### Tph1−/− mice exhibited a milder extent of postoperative intra-abdominal adhesion formation induced by caecum rubbing

**Figure 1:**
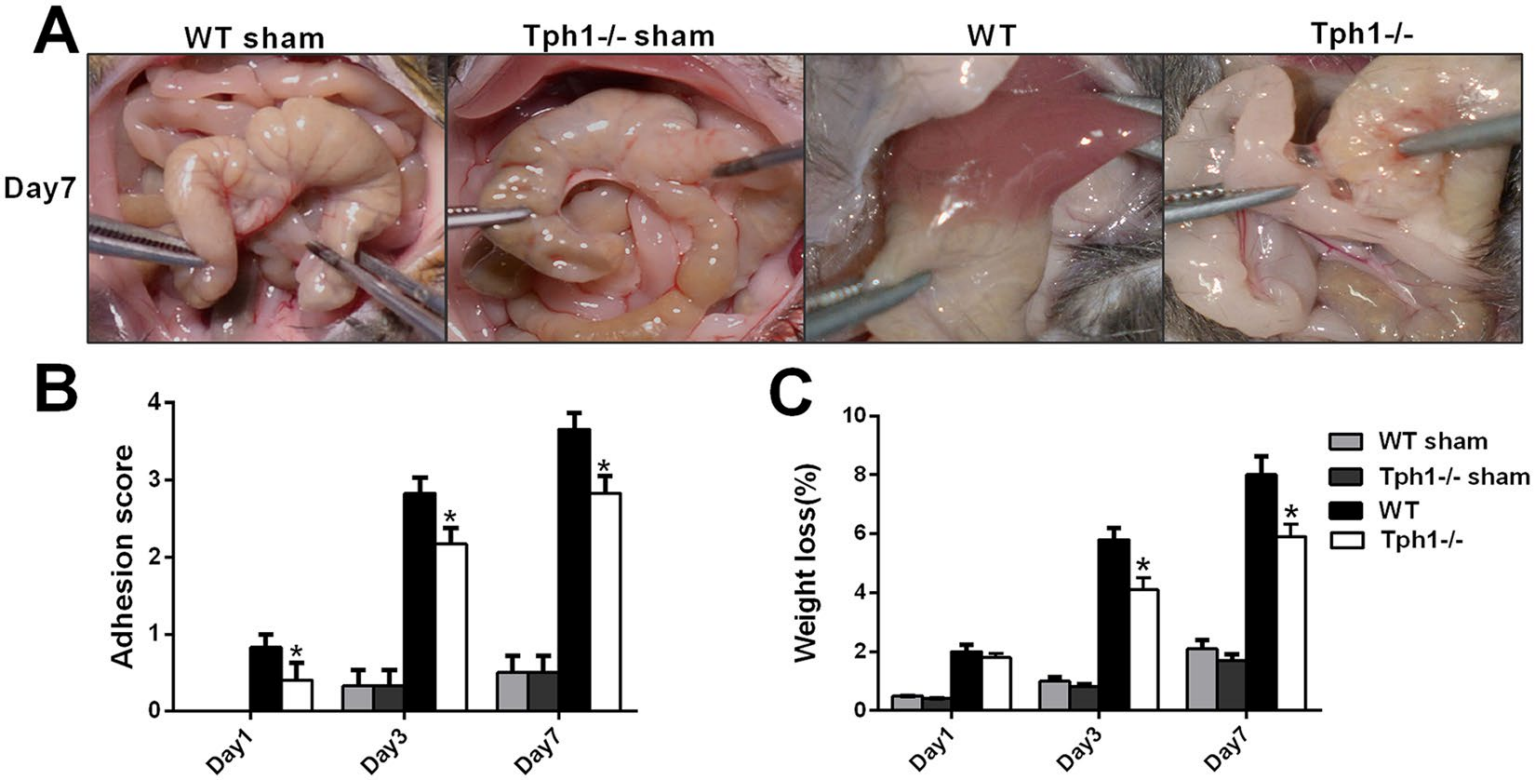
Tph1−/− mice exhibited a milder extent of postoperative intra-abdominal adhesion formation. Postoperative intra-abdominal adhesion formation was induced by caecum rubbing, and the mice were sacrificed on the 1^st^, 3^rd^ and 7^th^ days after the operation. (**A**) Representative images of intra-abdominal adhesions on postoperative day 7; (**B**) The intra-abdominal adhesion scores based on the Nair’s criteria on postoperative days 1, 3 and 7; (**C**) The percentage of weight loss on postoperative days 1, 3 and 7. n = 6, mean ± SEM, ^*^P < 0.05 vs. the WT group.

On days 1, 3 and 7 after caecum rubbing, the Tph1−/− and wild-type mice were sacrificed to observe the macroscopic adhesion conditions and assess the adhesion scores. The mice that underwent the caecum-rubbing procedure displayed varying degrees of adhesion that involved the peritoneum, omentum, epididymal fat pad, caecum, small intestine, liver and other structures. It was noteworthy that the Tph1−/− mice exhibited a milder extent of adhesion formation (Fig. 1A). Furthermore, the Tph1−/− mice received significantly lower scores than the WT mice on the 1^st^, 3^rd^ and 7^th^ days postoperatively (p < 0.05) (Fig. 1B). Additionally, the percentage of weight loss in the Tph1−/− group was less than that in the WT group on the 3^rd^ and 7^th^ day postoperatively (p < 0.05) (Fig. 1C).

### Deficiency of serotonin decreased the collagen deposition in postoperative intra-abdominal adhesions in mice

**Figure 2:**
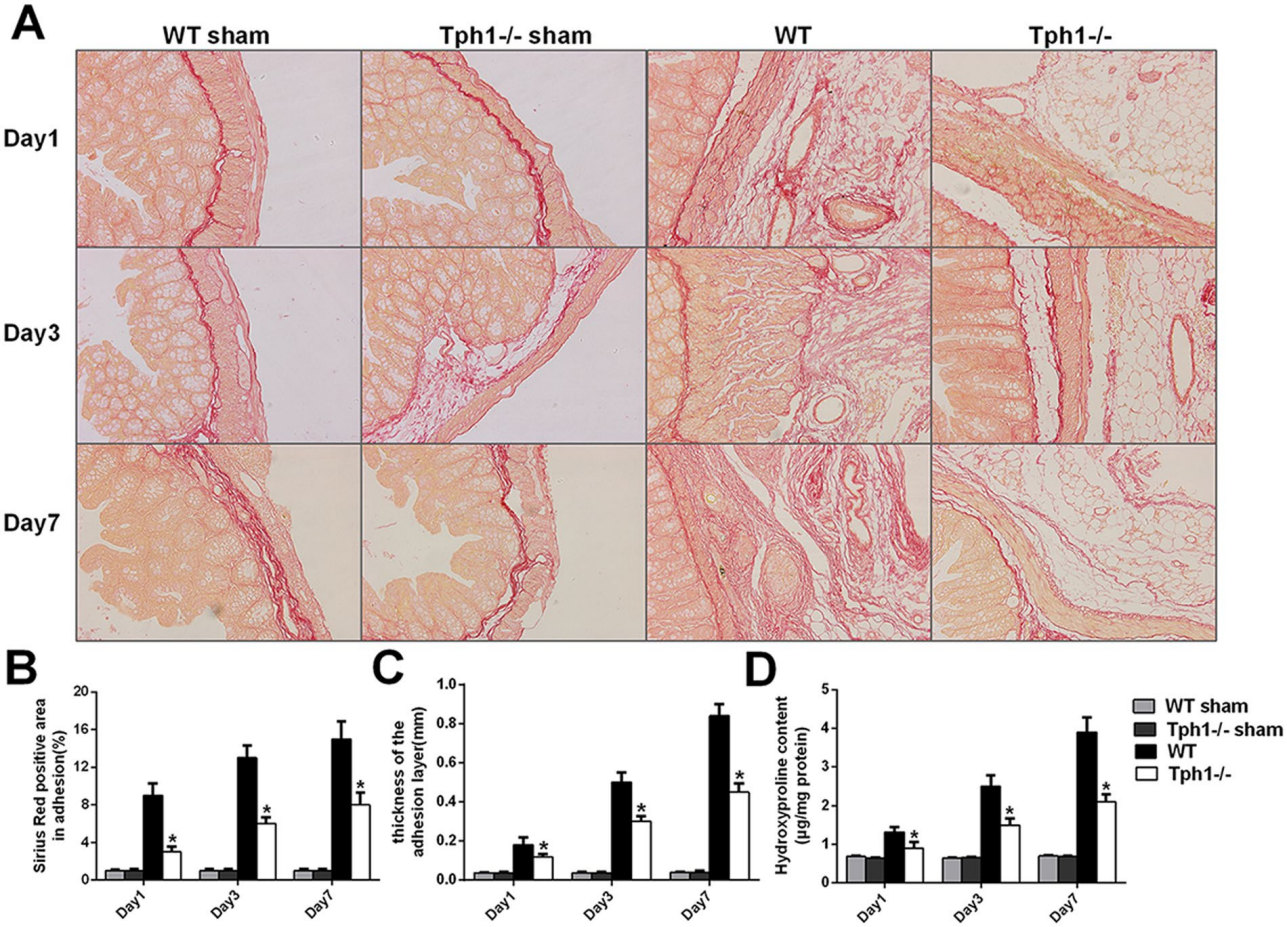
Deficiency of serotonin decreased collagen deposition in postoperative intra-abdominal adhesions in mice. Postoperative intra-abdominal adhesion formation was induced by caecum rubbing, and the mice were sacrificed on the 1^st^, 3^rd^ and 7^th^ days after the operation. The adhesive tissues in each group were removed and processed for analysis. (**A**) Representative images of picrosirius red staining showing collagen fibres in the adhesive tissues (magnification ×200); (**B**) Positively stained areas in the adhesive tissues using picrosirius red was calculated by ImagePro Plus 5.0 software; (**C**) The thickness of the collagen deposition in the adhesive tissues; (**D**) The hydroxyproline content in the adhesive tissues. n = 6, mean ± SEM, ^*^P < 0.05 vs. the WT group.

Picrosirius red staining was conducted to detect the collagen fibres in the adhesive tissues. The operation of caecum rubbing generated thick and dense collagen fibres in the abdominal cavity in the WT mice. Nevertheless, the Tph1−/− mice appeared to have thinner and looser collagen fibres ((Fig. 2A). The percentage of the area that stained positive with the picrosirius red stain area in the adhesive tissues of the Tph1−/− mice was significantly lower than that in the WT mice on the 1^st^, 3^rd^ and 7^th^ days postoperatively (p < 0.05) (Fig. 2B). The thickness of the adhesion layers was less in the Tph1−/− group in contrast to the WT group (p < 0.05) (Fig. 2C). Hydroxyproline is a type of characteristic amino acid in collagen that can reflect the level of collagen expression. The hydroxyproline contents were significantly increased after caecum rubbing, and the mice in the Tph1−/− group dramatically exhibited lower levels of hydroxyproline than did the mice in the WT group (p < 0.05) (Fig. 2D). The results indicated that the deficiency of serotonin decreased collagen deposition in the adhesive tissues.

### Deficiency of serotonin attenuated inflammation in postoperative intra-abdominal adhesion formation in mice

**Figure 3:**
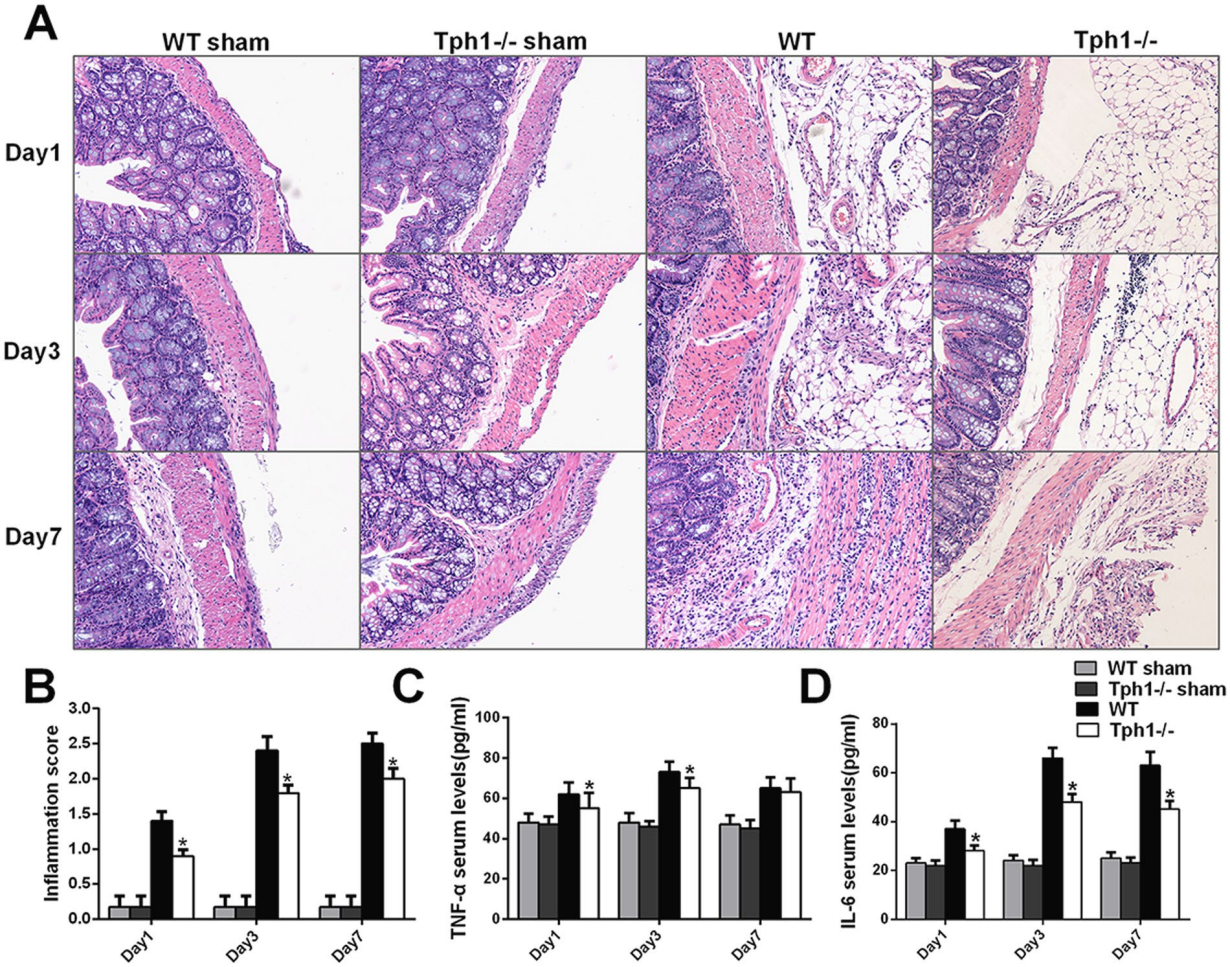
Deficiency of serotonin attenuated inflammation in postoperative intra-abdominal adhesion formation in mice. Postoperative intra-abdominal adhesion formation was induced by caecum rubbing, and the mice were sacrificed on the 1^st^, 3^rd^ and 7^th^ days after the operation. The adhesive tissues and blood samples in each group were harvested for analysis. (**A**) Representative images of haematoxylin-eosin staining of adhesive tissues (magnification ×200); (**B**) The inflammation scores were based on inflammatory cell infiltration and microabscess formation in the adhesive tissues; The levels of serum (**C**) TNF-α and (**D**) IL-6; n = 6, mean ± SEM, ^*^P < 0.05 vs. the WT group.

To determine the role of serotonin in the inflammatory response, the HE staining of the adhesive tissues and serum inflammatory cytokines were detected. The WT mice that underwent caecum rubbing exhibited massive inflammatory cell infiltration, including giant cells, lymphocytes, plasma cells, eosinophils and neutrophils, and even microabscess formation in the adhesive tissues. In contrast, the Tph1−/− mice only showed a mild inflammatory response that involved giant cells, lymphocytes, plasma cells, and a small number of eosinophils and neutrophils (Fig. 3A). The inflammation scoring based on the inflammatory cell infiltration and microabscess formation verified that the Tph1−/− group mice achieved markedly lower scores compared with the WT group on postoperative days 1, 3 and 7 (p < 0.05) (Fig. 3B). TNF-α and IL-6 are the initial and pivotal inflammatory cytokines that trigger the inflammatory cascades and promote postoperative intra-abdominal adhesion formation. A significant increase in serum TNF-α and IL-6 was observed after the cecum rubbing operation (p < 0.05). Additionally, the Tph1−/− group had significantly lower levels of serum TNF-α and IL-6 compared with the WT group (p < 0.05) (Fig. 3C,D).

### Deficiency of serotonin alleviated oxidative stress in postoperative intra-abdominal adhesion formation in mice

**Figure 4:**
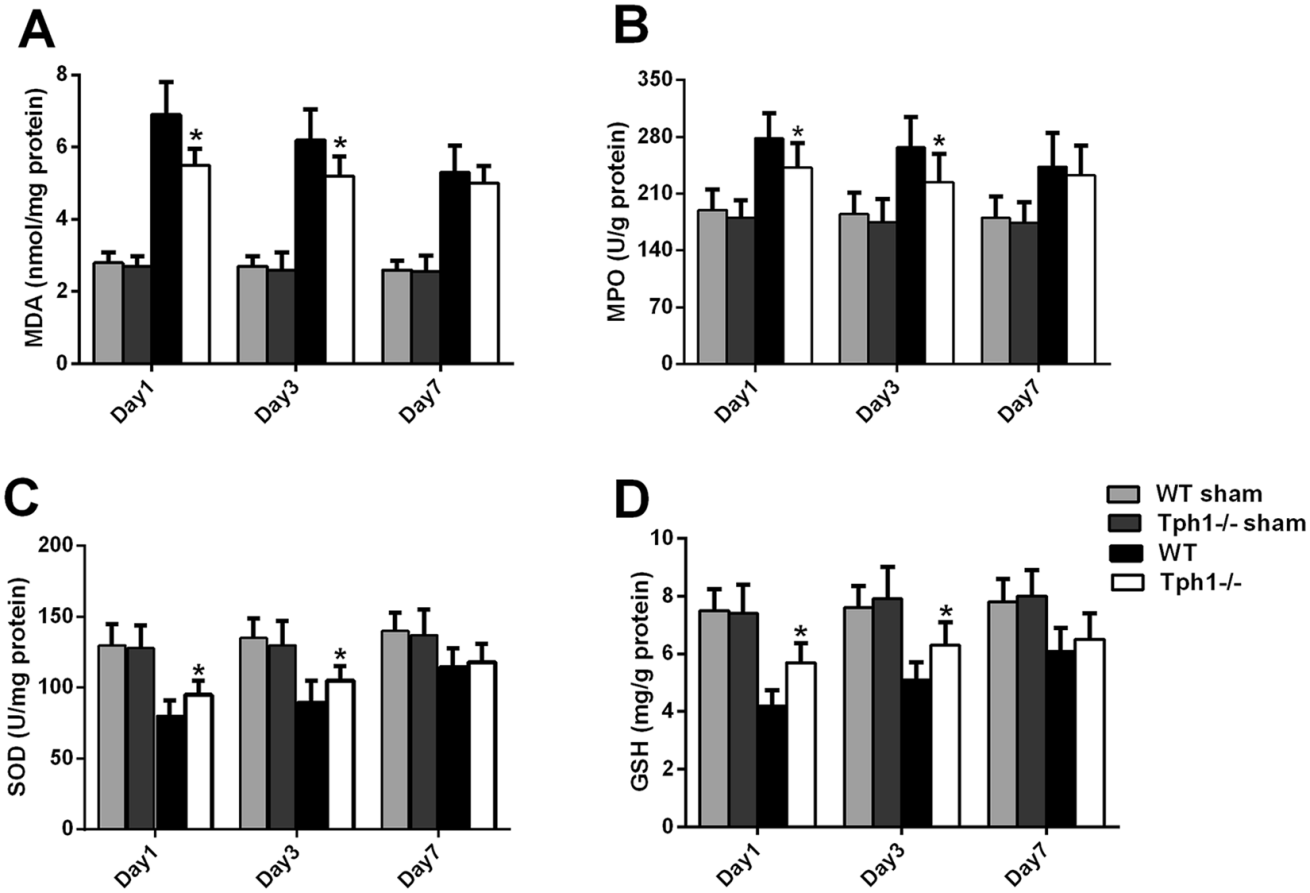
Deficiency of serotonin alleviated oxidative stress in postoperative intra-abdominal adhesion formation in mice. Postoperative intra-abdominal adhesion formation was induced by caecum rubbing, and the mice were sacrificed on the 1^st^, 3^rd^ and 7^th^ days after the operation. The adhesive tissues in each group were harvested to evaluate the oxidative stress. The levels of (**A**) MDA, (**B**) MPO, (**C**) SOD and (**D**) GSH in the adhesive tissues; n = 6, mean ± SEM, ^*^P < 0.05 vs. the WT group.

Oxidative stress participates in postoperative intra-abdominal adhesion formation with its direct damage to reactive oxygen species and indirect function of aggravating inflammation^31^. The Tph1−/− group mice had lower levels of MDA and MPO compared with the WT group (p < 0.05) (Fig. 4A,B). The protective indicators, SOD and GSH, were significantly decreased after the creation of the intra-abdominal adhesion model; however, the deficiency of serotonin exhibited certain degrees of reversal (p < 0.05) (Fig. 4C,D).

### Deficiency of serotonin relieved the disorders of the fibrinolytic system in postoperative intra-abdominal adhesion formation in mice

**Figure 5:**
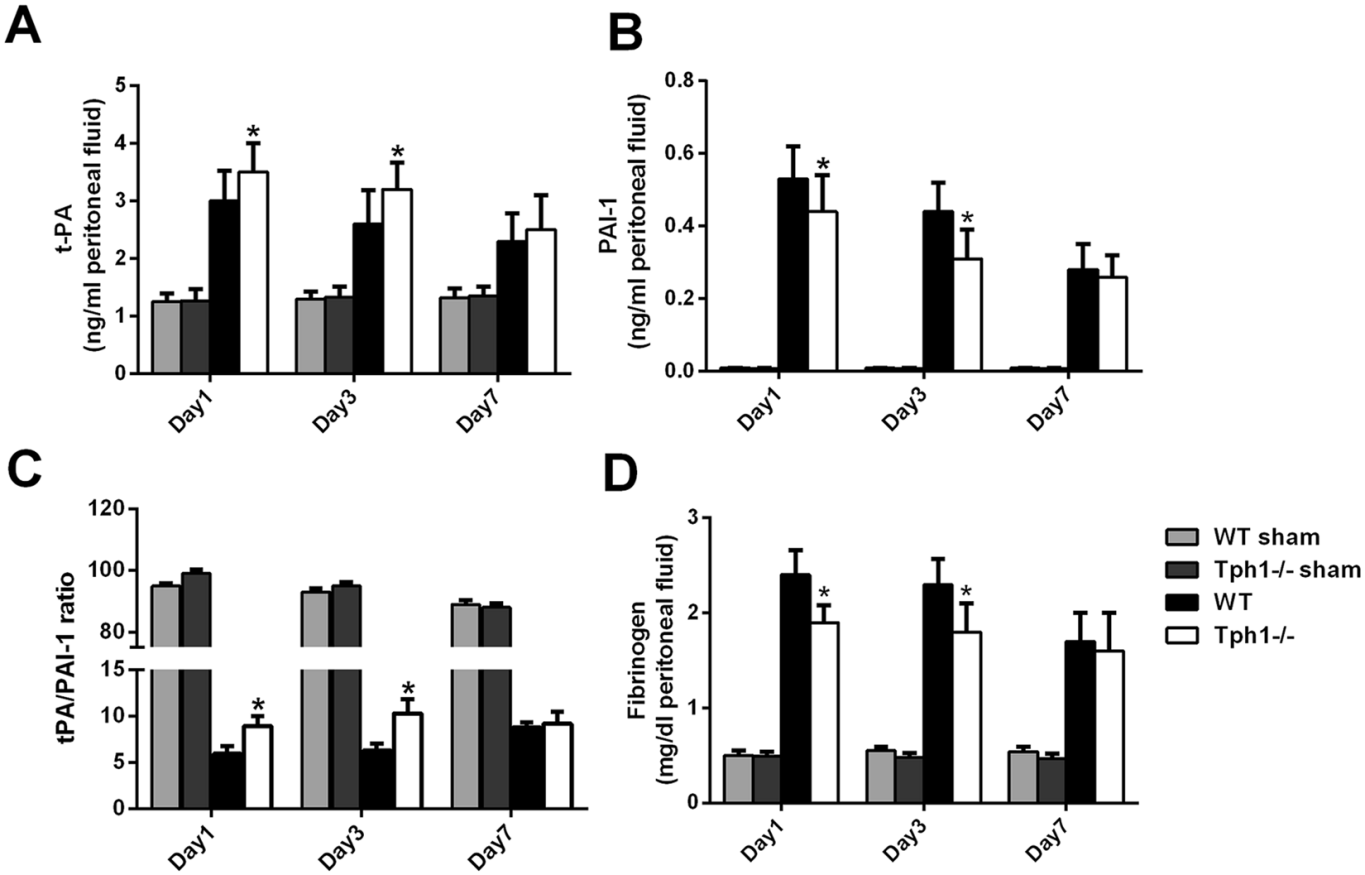
Deficiency of serotonin relieved the disorder of the fibrinolytic system in postoperative intra-abdominal adhesion formation in mice. Postoperative intra-abdominal adhesion formation was induced by caecum rubbing, and the mice were sacrificed on the 1^st^, 3^rd^ and 7^th^ days after the operation. The peritoneal fluids were harvested to evaluate the fibrinolytic system. The levels of (**A**) t-PA and (**B**) PAI-1 in the peritoneal fluids; (**C**) t-PA/PAI-1ratio in the peritoneal fluids; (**D**) The fibrinogen content in the peritoneal fluids; n = 6, mean ± SEM, ^*^P < 0.05 vs. the WT group.

The levels of t-PA in the peritoneal fluids were increased after adhesion modelling occurred. However, the Tph1−/− group mice had higher levels of t-PA compared with the WT group mice on postoperative days 1 and 3 (p < 0.05). The PAI-1, the t-PA antagonist, exhibited the opposite changes (Fig. 5A,B). The ratio of t-PA/PAI-1 was higher in the Tph1−/− group than in the WT group (p < 0.05) (Fig. 5C). The fibrinogen levels in the peritoneal fluids were increased after the caecum-rubbing procedure. The Tph1−/− group mice exhibited lower levels of fibrinogen compared with the WT group on the 1^st^ and 3^rd^ days postoperatively (p < 0.05) (Fig. 5D).

### Serotonin accelerated angiogenesis in postoperative intra-abdominal adhesion formation by up-regulating VEGF expression

**Figure 6:**
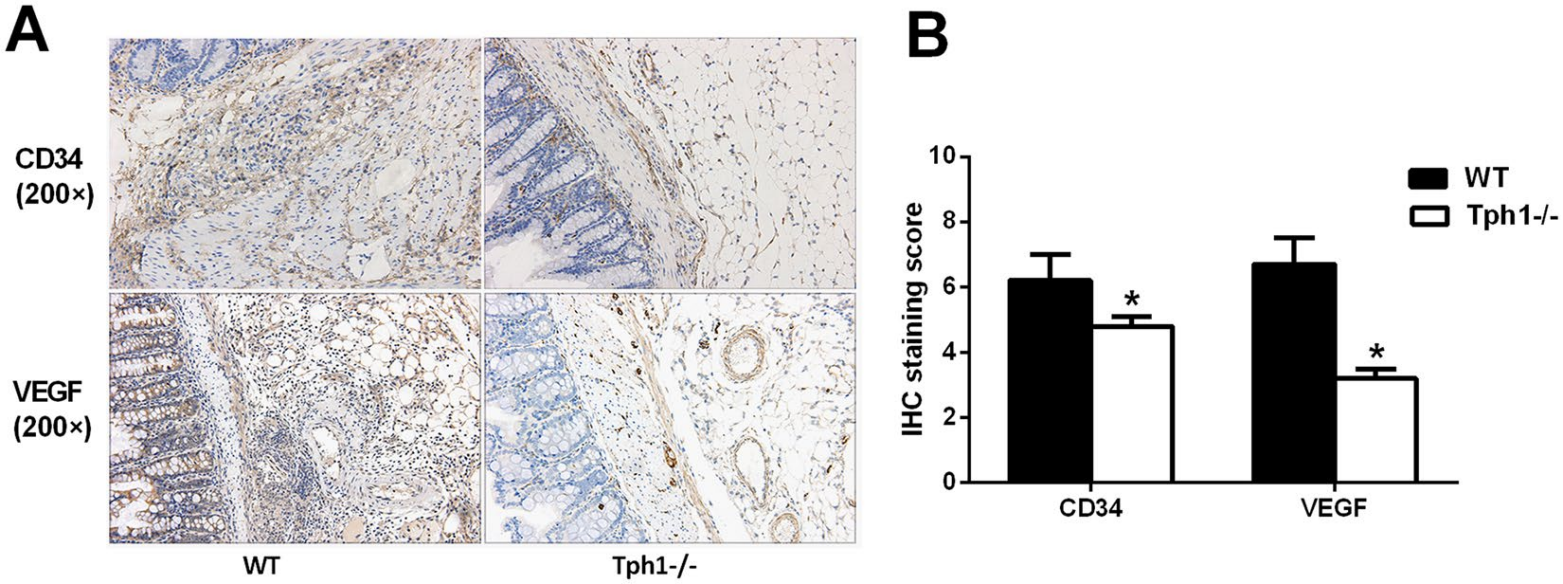
Serotonin accelerated angiogenesis in postoperative intra-abdominal adhesions by up-regulating VEGF expression. Postoperative intra-abdominal adhesion formation was induced by caecum rubbing, and the mice were sacrificed on the 7^th^ day after the operation. The adhesive tissues were harvested to detect the angiogenesis. (**A,B**) Immunohistochemistry for evaluating the levels of CD34 and VEGF in the adhesive tissues (magnification ×200); n = 6, mean ± SEM, ^*^P < 0.05 vs. the WT group.

CD34 is a marker of vascular endothelial cells and VEGF plays a pivotal role in angiogenesis. Immunohistochemistry verified that the expression levels of CD34 and VEGF in the Tph1−/− mice were lower than those in the WT mice, which was also confirmed by semi-quantitation (p < 0.05) (Fig. 6A,B). The results certified that serotonin accelerated angiogenesis in postoperative intra-abdominal adhesion formation by up-regulating the VEGF expression.

### Serotonin facilitated postoperative intra-abdominal adhesion formation via the 5-HT_2B_ receptor and up-regulating TGF-β1 expression

**Figure 7:**
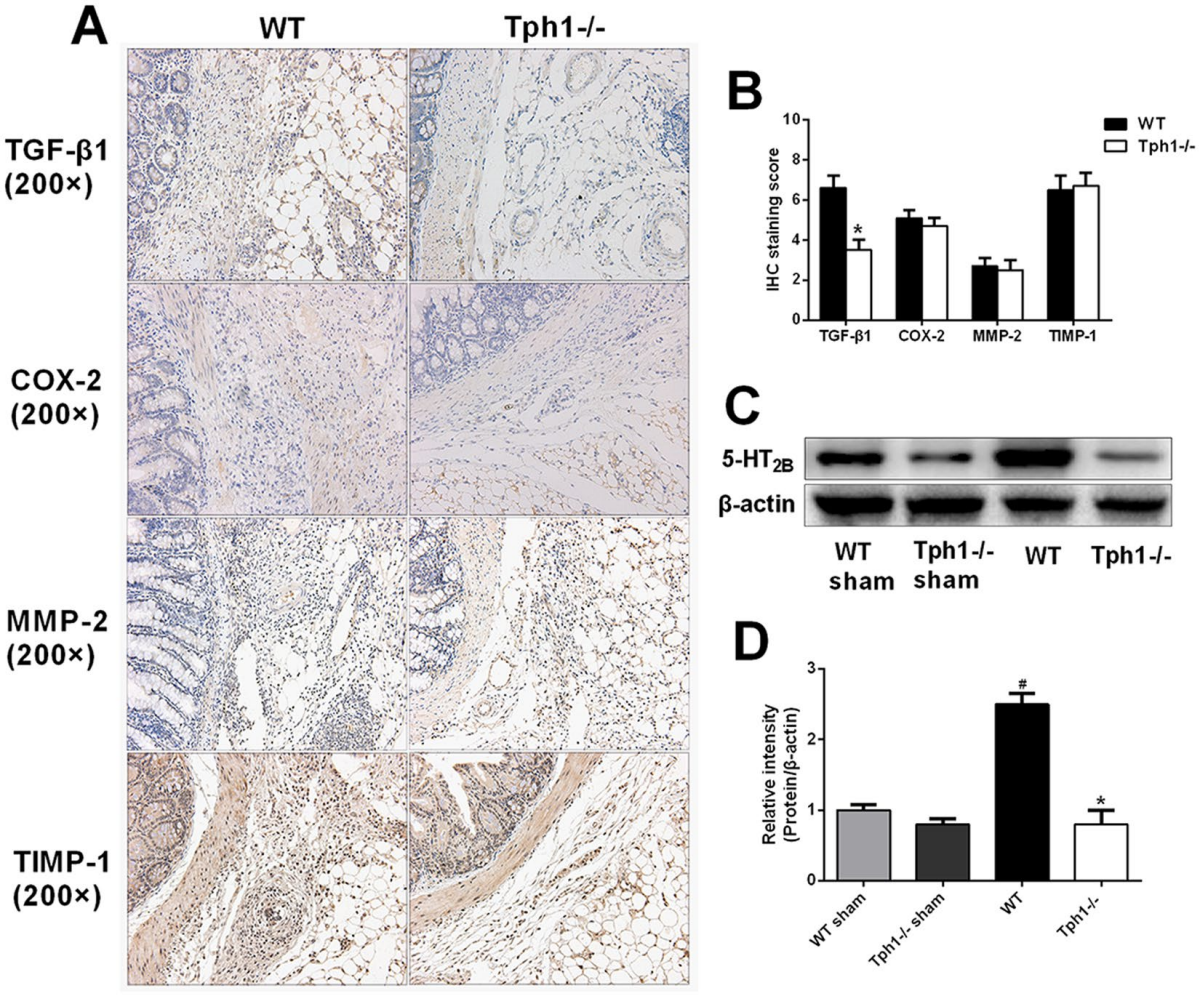
Serotonin facilitated postoperative intra-abdominal adhesion formation by up-regulating TGF-β1 expression. Postoperative intra-abdominal adhesion formation was induced by caecum rubbing, and the mice were sacrificed on the 7^th^ day after the operation. The adhesive tissues were harvested. (**A,B**) Immunohistochemistry was performed to evaluate the levels of TGF-β1, COX-2, TIMP-1 and MMP-2 in the adhesive tissues (magnification ×200); (**C,D**) Western blot analysis was conducted to evaluate the levels of 5-HT_2B_ receptor in the adhesive tissues; n = 6, mean ± SEM, ^#^P < 0.05 vs. the WT sham group, ^*^P < 0.05 vs. the WT group.

To study the potential mechanisms of serotonin in facilitating postoperative intra-abdominal adhesion formation, the related proteins including TGF-β1, COX-2, MMP-2 and TIMP-1 were detected. Immunohistochemistry and its semi-quantitation proved that the Tph1−/− group had a lower level of TGF-β1 in contrast to the WT group (Fig. 7A,B). Additionally, western blot analysis also revealed that the 5-HT_2B_ receptor was more highly expressed in the WT mice compared with the Tph1−/− mice (Fig. 7C,D).

### Inhibition of serotonin synthesis alleviated postoperative intra-abdominal adhesion formation in mice

To validate the role of serotonin in adhesion formation, 5-hydroxytryptophan (5-HTP, the precursor of serotonin) was applied to replenish serum serotonin in the Tph1−/− mice. The reloading of serotonin significantly increased the adhesion scores and weight loss (p < 0.05). Meanwhile, 5-HTP pretreatment aggravated the collagen fibre deposition and was associated with more serious inflammation than the Tph1−/− group mice (p < 0.05).

**Figure 8:**
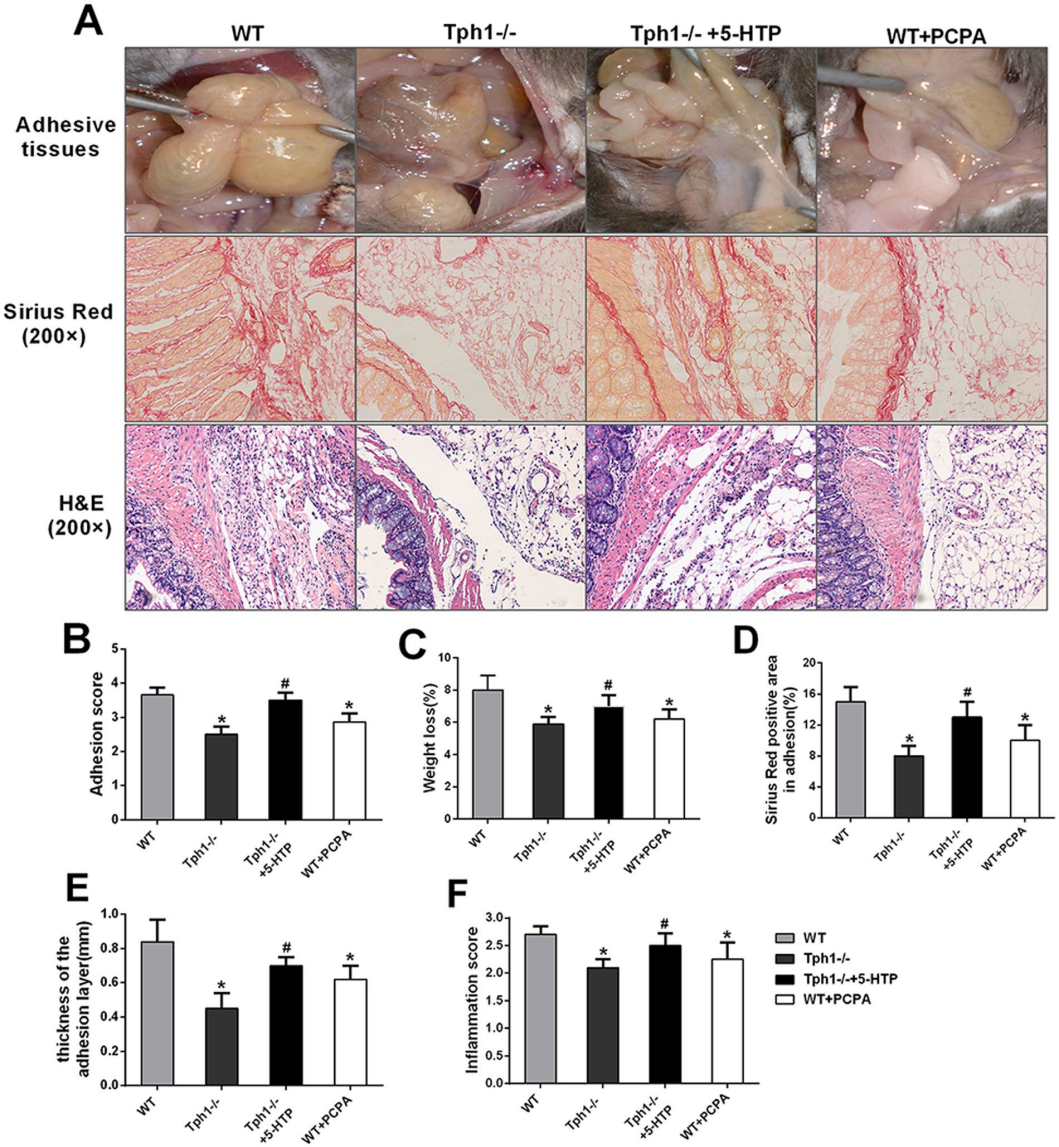
Inhibition of serotonin synthesis alleviated postoperative intra-abdominal adhesion formation in mice. The WT mice were pretreated with PCPA and the Tph−/− mice were pretreated 3 days before modelling. Postoperative intra-abdominal adhesion formation was induced by caecum rubbing, and the mice were sacrificed on the 7^th^ day after the operation to detect the severity of adhesion formation. (**A**) Representative images of adhesive bands, picrosirius red staining and haematoxylin-eosin staining of adhesive tissues; (**B**) Representative intra-abdominal adhesion scores based on the Nair, s criteria; (**C**) Percentage of weight loss, (**D**) Positive area according to picrosirius red staining; (**E**) Thickness of the collagen deposition and (**F**) Inflammation scores were assessed in the adhesive tissues; n = 6, mean ± SEM, ^*^P < 0.05 vs. the WT sham group, ^#^P < 0.05 vs. the Tph1−/− sham group.

To explore whether the intervention of serotonin synthesis could alleviate postoperative intra-abdominal adhesion formation, p-chlorophenylalanine (PCPA,the Tph1 inhibitor) was pretreated in the WT mice. The WT + PCPA group mice showed a milder extent of adhesion formation, lower adhesion score and weight loss compared with the WT group (p < 0.05) (Fig. 8A–C). The picrosirius red staining proved that the PCPA pretreatment significantly decreased the collagen fibre deposition and thickness of the adhesion layers (p < 0.05) (Fig. 8D,E). In addition, PCPA markedly quenched the inflammatory response in the adhesive tissues (Fig. 8F). The results indicated that the suppression of serotonin synthesis might be a new method for prevention of postoperative intra-abdominal adhesion formation.

## Discussion

**Figure 9:**
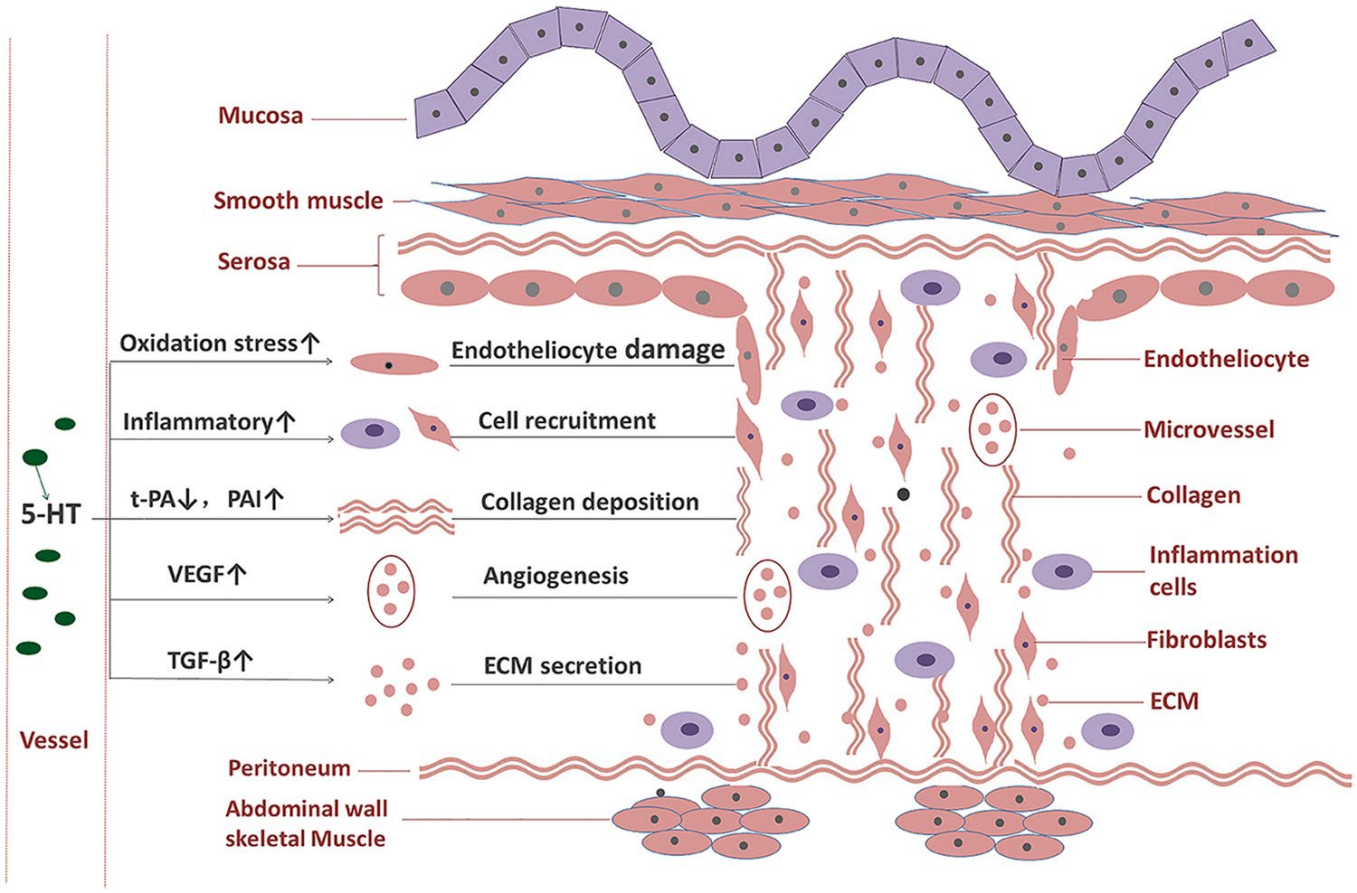
Peripheral serotonin regulated postoperative intra-abdominal adhesion formation in mice. After the surgery, serum serotonin was substantially released. Serotonin facilitated the following functions: recruitment of inflammatory cells and fibroblasts to the injury site, damage related to oxidative stress to the endotheliocyte, and the disorder of the fibrinolytic system, all of which resulted in collagen deposition, angiogenesis via the up-regulation of VEGF expression, and the secretion of extracellular matrix (ECM) via the up-regulation of TGF-β1 expression. The above mechanisms contributed to postoperative intra-abdominal adhesion formation.

In the present study, the model of postoperative intra-abdominal adhesions was established by caecum rubbing in the WT and Tph1−/− mice. We verified that serotonin regulated postoperative intra-abdominal adhesion formation in mice. The potential mechanisms might be related to serotonin’s role in facilitating inflammation, oxidative stress, disorder of the fibrinolytic system, angiogenesis, and TGF-β1 expression (Fig. 9). In addition, the exogenous antagonist of the serotonin synthesis enzyme significantly alleviated postoperative intra-abdominal adhesion formation in mice.

Inflammation on a large scale occurs when the peritoneum is injured^32^. Inflammatory cells, such as giant cells, lymphocytes, and plasma cells, as well as fibroblasts, are recruited to the injured area^33^. Moreover, the inflammatory cytokines are substantially released. These processes lead to the development of fibrous adhesions^4^. In addition, the oxidative stress and nitrative stress play important roles in adhesion formation^34^. The ROS, MPO and NO directly damage the mesothelial cells and suppress fibrinolytic activity in the injured peritoneum^31^. Several studies have demonstrated that serotonin has pro-inflammatory and pro-oxidative functions in many diseases^22, 24^. Our study verified that the deficiency of serotonin markedly decreased inflammation scores and the levels of TNF-α and IL-6. Moreover, the oxidative stress indicators, MDA and MPO were suppressed in the tissues with adhesions. The results demonstrated that serotonin might aggravate postoperative intra-abdominal adhesions in mice by facilitating inflammation and oxidative stress.

The fibrinolytic system is a complex and dynamic variation in the process of intra-abdominal adhesion formation. Fibrinous exudation is an essential physiological activity of tissue repair. However, the persistent fibrin deposition promotes fibroblast recruitment, new blood vessel formation and tissue remodelling^10^. Plasminogen activators (PAs) including t-PA and u-PA are serine proteases that convert plasminogen to plasmin. Both t-PA and u-PA can be inhibited by PAI-1^6^. The previous studies showed that the levels of t-PA and PAI-1 in the peritoneal fluids were markedly elevated after the surgery^35, 36^. Furthermore, it has been verified that serotonin content has a positive correlation with the PAI-1 levels in a concentration- and time-dependent manner in rat aortic endothelial cells^37^. In this study, we discovered that the concentrations of t-PA and PAI-1 in peritoneal fluid were markedly increased after the caecum-rubbing operation. However, the deficiency of serotonin significantly increased the t-PA level and t-PA/PAI-1 ratio and reduced the PAI-1 and fibrinogen contents in peritoneal fluid compared with the WT mice in the adhesion model on postoperative days 1 and 3. The study proved that serotonin might facilitate the disorder of the fibrinolytic system in postoperative intra-abdominal adhesions.

Angiogenesis is an important pathological process in adhesion formation^38^. CD34 is a marker of vascular endothelial cells, and VEGF plays a pivotal role in angiogenesis. One study demonstrated that VEGF is up-regulated in the adhesion formation^39^. Anti-angiogenesis drugs significantly alleviated postoperative intra-abdominal adhesion formation^40, 41^. Furthermore, the previous studies indicated that serotonin facilitates angiogenesis by accelerating VEGF expression in several diseases^42, 43^. In our study, we verified that a deficiency of serotonin could markedly decrease the elevation of CD34 and VEGF expressions in the adhesion model. Additionally, studies have shown that the TGF-β1 is over-expressed in adhesions^44, 45^.

TGF-β1 is responsible for the secretion of extracellular matrix (ECM) proteins, tissue remodelling and angiogenesis in peritoneal fibroblasts^12, 46^. COX-2, the rate-limiting enzyme of prostaglandin (PG) synthesis, is increased in adhesive tissues^47^. The MMPs, known as zinc-dependent proteolytic enzymes, can degrade specific proteins in the extracellular matrix^48^. The MMPs are decreased, while the TIMPs, inhibitors of MMPs, are highly expressed in adhesive tissues^49^. It has been verified that peripheral serotonin facilitates tissue remodelling and fibrosis by up-regulating TGF-β1 expression. Moreover, serotonin, which binds to its 5-HT_2A_ receptor, enhanced the secretion of TGF-β1 and MMPs in cardiac fibroblasts^50^. In addition, a significant positive correlation of 5-HT_2B_ receptor expression with MMPs was observed in canine dilated cardiomyopathy^51^. Furthermore, serotonin has a close relationship with COX-2 expression^52^. In the present study, we discovered that the expressions of TGF-β1, COX-2 and TIMPs were markedly increased after the caecum-rubbing operation. There were no differences in the levels of COX-2, MMPs and TIMPs between the WT mice and Tph1−/− mice. However, the serotonin knockout mice exhibited significantly lower levels of TGF-β1 in the adhesive tissues compared with the WT mice. Serotonin might exert its role of facilitating adhesions by up-regulating TGF-β1 expression.

The previous study showed that 5-HT_2_ receptors blockade decreased the formation of intra-abdominal adhesions in a dose-dependent manner in a rat caecum serosal denuding procedure^53^. Clara Dees *et al*. verified that 5-HT/5-HT_2B_ signalling links vascular damage and platelet activation to tissue remodelling^25^. In the current study, we discovered that 5-HT_2B_ receptors were highly expressed in the WT mice after the caecum-rubbing operation, and maintained a low level in the Tph1−/− mice. Serotonin might facilitate postoperative intra-abdominal adhesion formation via its 5-HT_2B_ receptor.

This study’s findings indicated that serotonin was involved in postoperative intra-abdominal adhesion formation in mice. Serotonin facilitated inflammation, oxidative stress, disorders of the fibrinolytic system, angiogenesis, and TGF-β1 expression via the 5-HT_2B_ receptor in the adhesive tissues. Moreover, PCPA pretreatment could alleviate adhesion formation. Serotonin may be a new target for adhesion prevention and therapy. However, the present study was only performed in mice. Further *in vitro* studies are needed to investigat deeper mechanisms. Moreover, prospective clinical studies are needed to evaluate whether the level of 5-HT relates to the process of postoperative intra-abdominal adhesion formation.

## Materials and Methods

### Experimental animals

The research was conducted using male wild-type C57BL/6 J mice (the Experimental Animal Center of Xi’an Jiaotong University) and male Tph1−/− mice (4–5 weeks of age, 20–26 g). The Tph1−/− mice were described previously^16^. The mice were fed in the Animal Feeding Center of Xi’an Jiaotong University Health Science Centre. The mice were housed (5 mice per cage) at 23 °C, 50% humidity, 12 h of light daily and free access to food and water. All animals were euthanized using isoflurane gas for tissue collection. The animal protocol was designed to minimize pain and discomfort to the animals. All animal experiments were performed in accordance with the guidelines of the China Council on Animal Care and Use. All animal procedures performed in this study were reviewed, approved, and supervised by the Institutional Animal Care and Use Committee of the Ethics Committee of Xi’an Jiaotong University Health Science Centre, China.

### Experimental design

Mice were randomly allocated into the following groups: (1) WT sham group: wild-type C57BL/6 mice undergoing sham operation; (2) Tph1−/− sham group: Tph1 knockout mice undergoing sham operation; (3) WT group: wild-type C57BL/6 mice undergoing the caecum rubbing model; (4) Tph1−/− group: Tph1 knockout mice undergoing the caecum rubbing model; (5) Tph1−/− + 5HTP group: Tph1 knockout mice pretreated with 5-hydroxytryptophan (5-HTP, precursor of 5-HT, replenishing the serum serotonin content, 75 mg/kg/day for 3 days by subcutaneous injection) before the caecum rubbing model as previously reported^54, 55^; (6) WT + PCPA group: wild-type C57BL/6 mice pretreated with p-chlorophenylalanine (PCPA, Tph1-inhibitor, 150 mg/kg/day for 3 days by subcutaneous injection) before the caecum rubbing model as previously reported^55, 56^. The experiment was designed (6 mice per group) to assess adhesion formation on postoperative days 1, 3 and 7. To assess the inflammatory factors, mouse blood samples were collected from the periorbital plexus after the mice were anaesthetized with isoflurane gas. Following blood collection, the mice were euthanized. The peritoneal fluids and adhesive tissues were obtained to assess the extent of the intra-abdominal adhesions and determine the potential mechanisms.

### Postoperative intra-abdominal adhesions model

The postoperative intra-abdominal adhesion model was induced by laparotomy with caecum rubbing, which was validated by previous studies^57, 58^. The mice fasted 12 h and were forbidden to drink for 6 h before the operation. The mice were anaesthetized with chloral hydrate (10 ml/kg, 4%). The abdomen was disinfected with iodine volts cotton swabs, and an approximate 1.5-cm midline abdominal incision was performed. The caecum was exposed and rubbed gently with 2 dry gauze pads on the ventral and dorsal surfaces until the shine was lost and haemorrhagic points became visible. Next, the caecum was placed back in its original place and closed in layers. The sham operation was conducted abdominal incision only, which was exposed for 3 minutes and subsequently closed in layers. The mice were given 30 ml/kg saline for recovery from anaesthesia and were returned to their cages after a full recovery.

### Adhesion scoring system

After euthanasia, the extent of the postoperative intra-abdominal adhesions was assessed according to the adhesion score criteria reported by Nair, *et al*.^59^ (Table S1). The score criteria were as follow: 0, no adhesion band; 1, one filmy adhesion band between the viscera or between the viscera and abdominal wall; 2, two thin bands between the viscera or between the viscera and abdominal wall; 3, more than two moderate bands between the viscera or between the viscera and the abdominal wall, or the whole intestine formed a mass without adhering to the abdominal wall; and 4, very thick adhesion band between the viscera and the abdominal wall. The scores were assessed in a blind manner by two researchers.

### Picrosirius red staining for collagen

On days 1, 3, and 7 following the caecum-rubbing modelling, the adhesive tissues were collected, fixed in 10% formalin at 4 °C for 48 h, and then embedded in the paraffin. Serial 5-μm-thick sections were obtained, and 0.1% picrosirius red (Sigma-Aldrich, St. Louis, MO, USA) was used for staining. The picrosirius red staining was evaluated by two researchers in a blind manner using a microscope and a representative field was chosen for application. The percentage of the adhesion area that stained positive using picrosirius red was calculated using ImagePro Plus 5.0 software.

### Hematoxylin and Eosin (HE) Staining and inflammation score

The 5-μm-thick sections were obtained from the paraffin. The hematoxylin and eosin (HE) staining of adhesive tissues was performed. Next, the histological changes were assessed in a blind manner by two researchers using a light microscope, and a representative field was chosen for application. The inflammation score was determined using the following classical criteria described previously^60^ (Table S2). 0, no inflammation; 1, mild inflammation: infiltration of giant cells, lymphocytes, and plasma cells; 2, moderate inflammation: infiltration of giant cells, plasma cells, eosinophils, and neutrophils; 3. severe inflammation: inflammatory cell infiltration and microabscess formation.

### Enzyme-linked immunosorbent assays (ELISA)

The levels of serum TNF-a, IL-6 as well as t-PA, PAI-1 and fibrinogen in the peritoneal fluid were measured using commercial ELISA kits according to the instructions of the manufacturer (Dakewe, Shenzhen, China).

### Measurement of adhesion oxidative stress

The concentrations of malonaldehyde (MDA), superoxide dismutase (SOD), myeloperoxidase (MPO) and glutathione (GSH) in the peritoneal adhesive tissues were measured as markers of oxidative stress. The adhesive tissues were homogenized, and measured using the activity assay kit (NanJing JianCheng Bioengineering Institute). All of the procedures were conducted according to the instructions of the manufacturer.

### Hydroxyproline determination

The content of hydroxyproline was measured using a Hydroxyproline Assay Kit (Sigma-Aldrich) according to the manufacturer’s instructions. The results were expressed in μg/mg of protein.

### Western blot analysis

The peritoneal adhesive tissues were collected and stored at −80 °C after the mice were sacrificed. The proteins were extracted from the adhesive tissues using RIPA lysis buffer. BCA protein assay kit was used for detecting the concentration of extracted proteins. The proteins were separated by 10% sodium dodecyl sulfatepolyacrylamide gel electrophoresis, and transferred onto poly-vinylidene difluoride (PVDF) membranes. The PVDF membranes were blocked with 5% skim milk and incubated with primary antibodies, anti-5-HT_2B_ antibody (bs-1892R, BIOSS, China, 1:500 dilution), and anti-β-actin antibody (sc-47778, Santa Cruz Biotechnology, USA, 1:1000 dilution). The membranes were incubated with secondary antibodies for 2 h at room temperature. The protein expressions were detected using a chemiluminescence system (BIO-RAD) according to the manufacturer’s specifications. The protein quantification was performed using ImageJ2x software. The results are expressed as relative intensity of protein/β-actin.

### Immunohistochemistry

The VEGF, CD34, TGF-β1, COX-2, TIMP-1 and MMP-2 expressions in peritoneal adhesive tissues were detected by immunohistochemical staining. The detailed procedures were conducted as previously described^61^. The primary antibodies anti- CD34 antibody (bs-9752R, BIOSS, China, 1:300 dilution), anti- VEGF antibody (bs-0279R, BIOSS, China, 1:300 dilution), anti-TGF-β1 antibody (sc-146, Santa Cruz Biotechnology, USA, 1:400 dilution), anti-COX-2 antibody (bs-10411R, BIOSS, China, 1:300 dilution), anti-TIMP-1 antibody (bs-4600R, BIOSS, China, 1:300 dilution) and anti-MMP-2 antibody (bs-4599R, BIOSS, China, 1:300 dilution) were incubated overnight at 4 °C, and a secondary antibody was incubated for 1 h at room temperature. The staining was assessed in a blind manner using a light microscope, and a representative field was chosen for as application. The semi-quantitation of immunohistochemical staining was performed, and the intensity was scored as follows: 0, negative; 1, mild; 2, moderate; and 3, severe. The extent of staining was assessed based on the percentage of positive cells and was scored as follows: 0, negative; 1, 1–25%; 2, 26–50%; 3,51–75%; and 4,76–100%. The staining scores were the sum of the intensity and extent scores, and ranged from 0 to 7. The final staining score was represented as the mean in each group.

### Statistical analysis

All the measurement data are expressed as the means ± SEM. The t-test or one way ANOVA was applied to analyse the differences between groups, using data statistics software SPSS 18.0. P < 0.05 represents a significant difference.

## Electronic supplementary material

Supplement
