# Supplementary material for: Peripheral serotonin regulates postoperative intra-abdominal adhesion formation in mice
Source: Sci Rep. 2017 Aug 30;7:10001. doi: 10.1038/s41598-017-10582-w (PMC5577130; doi:10.1038/s41598-017-10582-w)
Supplement: Supplementary file 1 — Supplement [file 41598_2017_10582_MOESM1_ESM.pdf]

Peripheral serotonin regulates the postoperative intra-abdominal adhesion formation in mice

Running title: Serotonin facilitates the intra-abdominal adhesion formation

Jianbin Bi[1,2], Simin Zhang[1], Zhaoqing Du[1,2], Jia Zhang[1,2], Yan Deng[1], Chang Liu[1,3*], Jingyao Zhang[1,3*]

Table SI. Nair's adhesion score.

Score	The criterions for Nair's adhesion score
0	No adhesion band
1	One filmy adhesion band between the viscera or between the viscera and abdominal wall
2	Two thin bands between the viscera or between the viscera and abdominal wall
3	More than two moderate bands between the viscera or between the viscera and the abdominal wall, or the whole intestine forms a mass without adhering to the abdominal wall
4	Very thick adhesion band between the viscera and the abdominal wall

Table S2. Adhesions' Inflammation score.

Score	The criterions for inflammation score of adhesions
0	No inflammation
1	Mild inflammation: infiltration of giant cells, lymphocytes, and plasma cells
2	Moderate inflammation: infiltration of giant cells, plasma cells, eosinophils, and neutrophils
3	Severe inflammation: inflammatory cell infiltration and microabscess formation
